# Supplementary material for: Annexin A1 as Neuroprotective Determinant for Blood-Brain Barrier Integrity in Neonatal Hypoxic-Ischemic Encephalopathy
Source: J Clin Med. 2019 Jan 24;8(2):137. doi: 10.3390/jcm8020137 (PMC6406389; doi:10.3390/jcm8020137)
Supplement: Supplementary file 1 [file jcm-08-00137-s001.pdf]

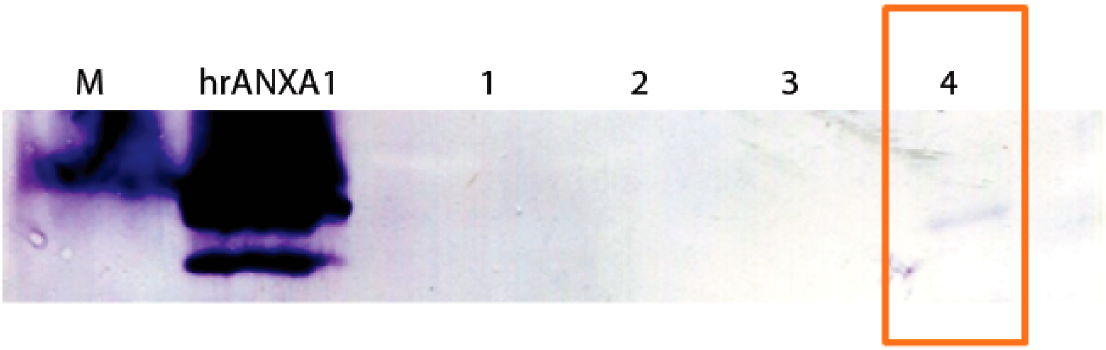

Figure S1. Western blot of MSC-EVs and negative controls for ANXA1. M: broad range protein marker (45 kDa); ANXA1: positive control 200 ng hrANXA1; 1: pure hPL, 2: pure hPL + 10,000× *g*, 10 minutes, 3: pure hPL +10,000× *g*, 10 min + 0.2 μM filtered, 4: MSC-EVs.

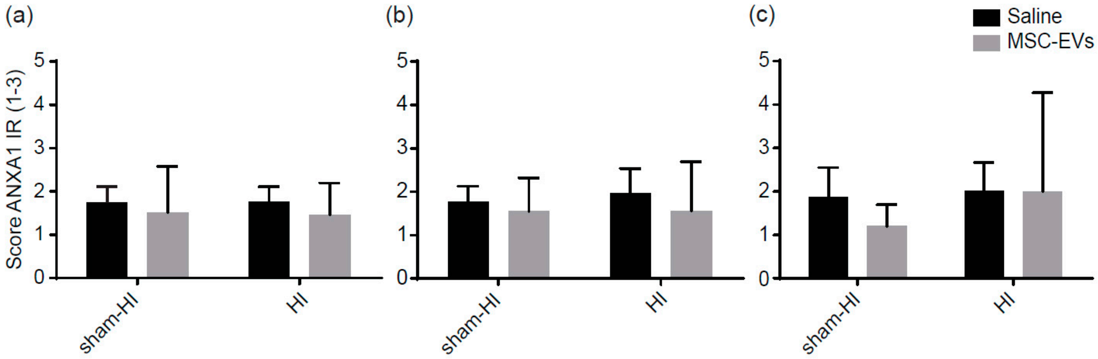

Figure S2. ANXA1 IR within (**a**) cerebral blood vessels, (**b**) ependyma and (**c**) microglia in response to MSC-EV treatment 7 days after HI or sham-HI.
